# Real-World Evidence: Multicenter Efficacy and Toxicity Analysis of Nintedanib With Docetaxel as Second-Line Treatment in Mexican Patients With Advanced Lung Adenocarcinoma

**DOI:** 10.1200/JGO.19.00330

**Published:** 2020-03-20

**Authors:** Jeronimo Rafael Rodríguez-Cid, Saul Campos-Gomez, Vanessa García-Montes, Manuel Magallanes-Maciel, Rodrigo Rafael Flores-Mariñelarena, Valeria Michelle Fernández-Garibay, Iván Romarico González-Espinoza, Juan Paulo Ceja-García, Juan Carlos Cázarez-Price, Luis Martínez-Barrera, Leopoldo Barriguete-Parra, Carlos Jose Zuloaga-Fernandez, Roberto Kuri-Exsome, David Suárez-García, Jorge Ignacio Gonzalez-Villanueva, Noé Flores-Anaya, Jose Antonio Acevedo-Delgado, Alma Magdalena Astorga-Ramos, Raquel Gerson-Cwilich, Alberto Villalobos-Prieto, Claudia Rodríguez-Silva, Maria Fernanda Noriega-Iriondo, Leticia Vázquez-Cortés, Eusebio Perales-Rodríguez, Alicia Acosta-Espinoza, Yareni Perez-Lozano, Daniel Capdeville-García, Jorge Arturo Alatorre-Alexander

**Affiliations:** ^1^ Department of Oncology, Instituto Nacional de Enfermedades Respiratorias Ismael Cosío Villegas, Mexico City, Mexico; ^2^ Department of Oncology, Centro Oncológico Estatal ISSEMYM, State of Mexico, Toluca de Lerdo, Mexico; ^3^ Department of Oncology, Hospital Español, Mexico City, Mexico; ^4^ Department of Oncology, Hospital Central Militar, Mexico City, Mexico; ^5^ Department of Internal Medicine, Fundación Clínica Médica Sur, Mexico City, Mexico; ^6^ School of Medicine, Monterrey Institute of Technology and Higher Education, Mexico City, Mexico; ^7^ Department of Oncology, Hospital Ángeles Puebla, Puebla, Mexico; ^8^ Department of Oncology, Hospital General ISSSTE La Paz, Baja California Sur, Mexico; ^9^ Department of Oncology, American British Cowdray Medical Center, Mexico City, Mexico; ^10^ Department of Oncology, Centro Oncológico de Chihuahua, Chihuahua, Mexico; ^11^ Department of Oncology, Oncología Privada Integral, Guadalajara, Mexico; ^12^ Department of Oncology, Hospital Aranda de la Parra, Guanajuato, Mexico; ^13^ Department of Oncology, Seguro Popular, León, Mexico; ^14^ Department of Oncology, Oncare Treatment Center, Monterrey, Mexico; ^15^ Department of Oncology, Medionco, Mexico City, Mexico; ^16^ Department of Oncology, Unidad de Cancerología, Jalisco, Mexico; ^17^ Department of Oncology, Sanatorio San José, Monterrey, Mexico; ^18^ Department of Oncology, Hospital Universitario Dr. José Eleuterio González, Monterrey, Mexico; ^19^ Department of Oncology, Centro Médico Hospital San José, Monterrey, Mexico; ^20^ Department of Oncology, Clínica Médica ARCOS, Morelia, Mexico; ^21^ Department of Oncology, Servicio de Salud Pemex, Reynosa, Mexico; ^22^ Department of Oncology, Hospital ISSSTE Mexicali, Baja California, Mexico; ^23^ Department of Oncology, Hospital Puebla, Puebla, Mexico

## Abstract

**PURPOSE:**

The LUME-Lung 1 study has brought consistent evidence of the effective use of nintedanib in lung adenocarcinoma as a second line of treatment; however, differences among ethnicities have been found in some studies.

**METHODS:**

This was a retrospective review among 21 medical centers of 150 patients with a confirmed diagnosis of lung adenocarcinoma, included in a compassionate use program of nintedanib from March 2014 to September 2015. The current study aimed to analyze the effectiveness of nintedanib in combination with docetaxel in the Mexican population, using progression-free survival rate and the best objective response to treatment by RECIST 1.1 as a surrogate of effectiveness. In addition, we examined the toxicity profile of our study population as a secondary end point.

**RESULTS:**

After exclusion criteria, only 99 patients met the criteria for enrollment in the current study. From the total study population, 53 patients (53.5%) were male and 46 (46.5%) were female, with an average age of 60 years and stage IV as the most prevalent clinical stage at the beginning of the compassionate use program. A total of 48 patients (48.5%) had partial response; 26 (26.3%), stable disease; 4 (4%), complete response; and 16 (16.2%), progression; and 5 (5%) were nonevaluable. We found a median progression-free survival of 5 months (95% CI, 4.3 to 5.7 months). The most common grade 3 or 4 adverse reactions were fatigue (14%) and diarrhea (13%).

**CONCLUSION:**

Nintedanib, as part of a chemotherapy regimen, is an effective option with an acceptable toxicity profile for advanced lung adenocarcinoma after first-line treatment progression.

## INTRODUCTION

Lung cancer represents the leading cause of cancer-related deaths worldwide, with nearly all patients facing disease progression during or after first-line therapy.^1-4^ Approximately 85% of patients with lung cancer are found to have a non–small-cell lung cancer (NSCLC) tumor histology, from which 50% are further classified as adenocarcinoma, followed by squamous cell carcinoma with 40%, and other less common subtypes such as large cell carcinoma and carcinoid tumor accounting for the rest.^5,6^

Despite the growing research involving NSCLC pathobiology and the development of molecular-targeted therapies, additional efforts to improve treatment must be implemented.^7^ Failure to achieve prolonged disease control is characterized by poor survival with second-line treatment with currently approved second-line agents such as docetaxel, gemcitabine, pemetrexed (for nonsquamous histologic types), and afatinib in specific patient groups, which rarely exceed a median survival of 8 months.^8,9,10^

It is widely acknowledged that angiogenesis is vital for the growth, progression, and metastatic dissemination of many solid tumor types.^11,12^ These mechanisms of neovascular formation are regulated mainly by the activation of signaling pathways associated with vascular endothelial growth factor (VEGF), fibroblast growth factor (FGF), and platelet-derived growth factor (PDGF).^13,14^ The limitations of VEGF-targeted therapies are primarily related to their toxicity and development of resistance by the activation of alternative proangiogenic pathways, including the overexpression of FGF-2, and signaling via PDGF ligands and receptors, tumor necrosis factor α, and placental growth factor.^11^

**CONTEXT**

**Key Objective**
Is the combination of docetaxel with nintedanib an effective second-line treatment option for patients with advanced lung adenocarcinoma?
**Knowledge Generated**
Nintedanib’s strong antiangiogenic effects after first-line treatment progression were demonstrated with a median progression-free survival of 5 months, consistent with previous phase III randomized trials. The toxicity profile associated with the use of docetaxel with nintedanib was acceptable, with diarrhea and fatigue being the most common grade 3 or 4 adverse reactions.
**Relevance**
Nintedanib, when used as part of a chemotherapy regimen, can be an adequate alternative for the treatment of advanced lung adenocarcinoma and for those who have faced disease progression after receiving other antiangiogenic agents as first-line therapy.

Nintedanib is a tyrosine kinase inhibitor with the ability to target 3 major angiogenesis pathways, and it is now considered a promising agent for multiple antiangiogenic antineoplastic therapies, including NSCLC adenocarcinoma.^6,15,16^ Because of its triple angiokinase inhibitor activity, nintedanib competitively binds ATP-binding sites within the PDGF receptor family, the FGF receptor family, and the VEGF receptor family, thus halting their signaling pathways and limiting angiogenic progression on multiple fronts.^11,17,18^ Recent publications have presented evidence of the strong antiangiogenic capacity of nintedanib with downstream effects on multiple cell types including endothelial cells, smooth cells, pericytes, and fibroblasts.^2,8,11,19,20^ In addition, because of its pharmacokinetic properties, nintedanib has minimal drug interactions, which permits its combination with other chemotherapy agents, improving its efficacy.^9,21,22^ Nintedanib has shown a good tolerability profile with grade ≥ 3 adverse events, related mainly to increases in transaminases and asthenia.^5,23^ However, phase I nintedanib trials have reported differences between Japanese and European patients, with greater liver-limiting toxicity at a lower dose in the Japanese population,^24-26^ resulting in a need for a more precise security profile among different ethnicities.

In a phase III, multinational, randomized, placebo-controlled clinical trial (LUME-Lung 1), nintedanib showed positive and additive results in combination with docetaxel, an antimitotic chemotherapeutic agent, in the treatment of patients with advanced lung adenocarcinoma, but no reported benefit was shown in patients with squamous histology.^5^ In addition, in the LUME-Lung 1, the median progression-free survival (PFS) of 3.4 months in the nintedanib plus docetaxel group was significantly prolonged compared with the PFS of 2.7 months in the docetaxel plus placebo group. The study also showed a good safety profile, with a similar frequency of adverse events to the prior treatments.^8,27^

After the previous study, the LUME-Lung 2 clinical trial was performed, which compared nintedanib plus pemetrexed versus pemetrexed and placebo in patients with advanced NSCLC after the failure of first-line chemotherapy. Results from this study showed a better PFS in the nintedanib with pemetrexed arm, but because the study was stopped prematurely, no data on overall survival (OS) were recorded.^8,11,28^ Recently, the NIS VARGADO study, which is currently ongoing, investigated the efficacy and tolerability of nintedanib plus docetaxel as second-line treatment in patients with advanced NSCLC after first-line chemotherapy and/or immunotherapy; the study showed that 58.3% of patients developed a partial response, a disease control rate of 83.3% with a median PFS of 5.5 months.^29^

A network meta-analysis published in 2017 analyzed data across multiple randomized controlled trials and evaluated the relative efficacy of nintedanib plus docetaxel compared with other second-line agents in patients with adenocarcinoma histology NSCLC.^30^ Regarding both OS and PFS analysis, there was a statistically significant advantage of nintedanib plus docetaxel over monotherapy with docetaxel, erlotinib, and gefitinib.^30^ Comparison between nintedanib plus docetaxel and the newer treatment option, ramucirumab plus docetaxel, presented a similar relative efficacy, shown by no difference in either OS or PFS.^30^, Interestingly, when the combination of nintedanib plus docetaxel was compared with nivolumab as monotherapy, programmed death-ligand 1 (PD-L1) expression levels showed an important impact on patient outcomes. In those with low PD-L1 expression levels, there was no difference in OS between nivolumab and nintedanib plus docetaxel. In addition, in this same group, PFS favored those treated with nintedanib plus docetaxel. However, when PD-L1 expression levels were higher, nivolumab offered a greater advantage in OS.^30^ These findings suggest that the large proportion of patients with low PD-L1 expression levels could potentially receive a greater benefit when treated with nintedanib plus docetaxel.^30^ As of today, precise cutoff levels for PD-L1 in different subsets of patients remain to be determined for making appropriate treatment decisions in the future.^30,31^

The current study aimed to analyze the effectiveness of nintedanib in combination with docetaxel in the Mexican population, using PFS rate and the best objective response to treatment as a surrogate of effectiveness. In addition, we examined the toxicity profile of our study population as a secondary end point.

## METHODS

This was a descriptive, observational, retrospective, multicenter study of patients with nintedanib compassionate program in combination with docetaxel. The study design was approved by Instituto Nacional de Enfermedades Respiratorias’ Institutional Ethics Board following the Declaration of Helsinki, Fortaleza Brazil 2013. The study population met the following criteria: patients older than 18 years of age with a confirmed histopathologic diagnosis of lung adenocarcinoma, relapse, or failure of 1 first-line prior chemotherapy, Eastern Cooperative Oncology Group (ECOG) 0-1 at the beginning of the second-line treatment, locally advanced and/or metastatic clinical stage at the beginning of the second-line treatment, and acceptance to participate in the nintedanib compassionate use program by informed consent between March 2014 and September 2015. Excluded were those with more than 1 prior chemotherapy regimen, a previous non–platinum-based chemotherapy regimen, active CNS metastases, therapeutic anticoagulation (with the exception of low-dose heparin) or antiplatelet therapy, a history of major thrombotic or clinically relevant major bleeding, and any contraindications for docetaxel therapy, and those without a complete medical record or lost to follow-up.

The following clinical and pathologic variables were reviewed: sex, age, smoking status, ECOG, TNM initial clinical stage, mutational status, first-line treatment, response to treatment, adverse effects associated with the use of nintedanib (evaluated by the Common Terminology Criteria for Adverse Events version 4.03),^33^ PFS, and response as per Response Evaluation Criteria in Solid Tumors, version 1.1 (RECIST 1.1)^34^ using computed tomography scan. SPSS software version 24.0 (IBM, Armonk, NY) was used for analysis. The variables were expressed as median values, together with total values and percentages. PFS was graphed using a Kaplan-Meier plot. The criterion for statistical significance was *P* < .05. All financially related issues from the study were absorbed by the investigation group.

## RESULTS

**FIG 1:**
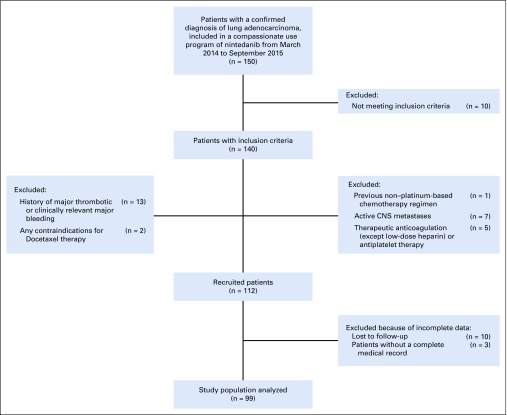
Flow diagram of the study population.

A total of 150 patients with a confirmed diagnosis of lung adenocarcinoma included in the compassionate program of nintedanib from March 2014 to September 2015 in 21 medical centers in Mexico were identified. After exclusion criteria, only 99 patients met the criteria for enrollment in the current study (Fig 1). From the total study population, 53 patients (53.5%) were male and 46 (46.5%) were female. The average age was 60 years (standard deviation, 12.36 years), with a range of 29 to 84 years. Fifty-three patients (53.5%) had a confirmed history of tobacco use (current or past smoker). At the beginning of treatment with nintedanib, 49 patients (49.5%) had an ECOG 0, and 50 patients (50.5%), an ECOG 1. First-line platinum-based chemotherapy was administered to all the patients; from the total study population, 7 patients (7.1%) had received previous therapy with bevacizumab and 38 (38.4%) had received a maintenance treatment. After the first-line treatment, 50 patients (50.5%) reported a partial response as the best response. Baseline characteristics of the study population are listed in Table 1.

**TABLE 1:**
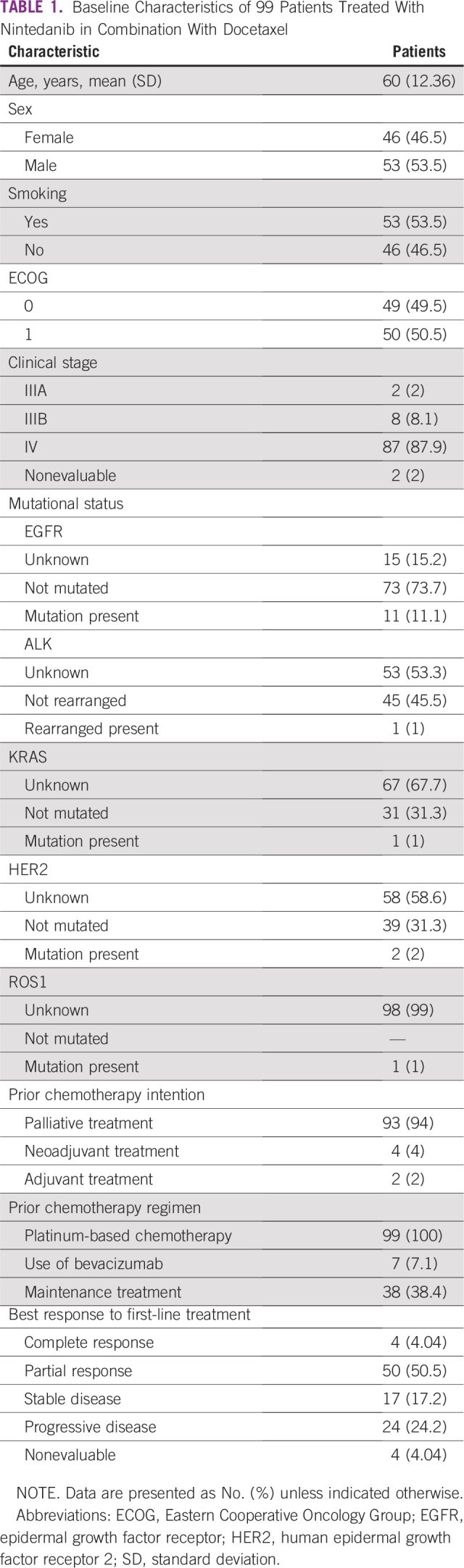
Baseline Characteristics of 99 Patients Treated With Nintedanib in Combination With Docetaxel

**FIG 2:**
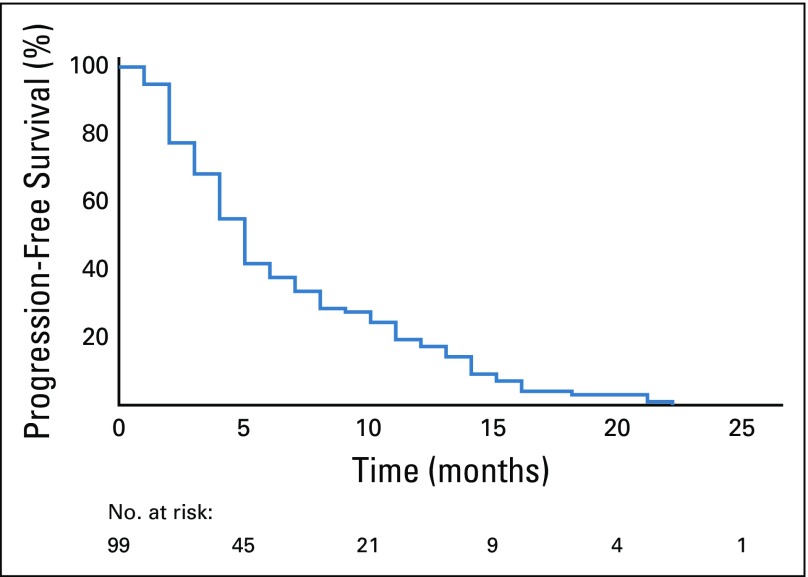
Progression-free survival.

After evaluating response to nintedanib therapy by RECIST 1.1, we found 48 patients (48.5%) with partial response, 26 (26.3%) with stable disease, 4 (4%) with complete response, 16 (16.2%) with progression, and 5 (5%) who were nonevaluable (Table 2). The median PFS was 5 months (95% CI, 4.3 to 5.7 months), as shown in Fig 2. The most common grade 3 or 4 adverse reactions were fatigue (14%), diarrhea (13%), hyporexia (7%), and neutropenia (7%), as listed in Table 3. Four patients had adverse events not related to nintedanib, including vena cava syndrome, pulmonary thromboembolism, bacterial pneumonia, and sepsis secondary to empyema.

**TABLE 2:**
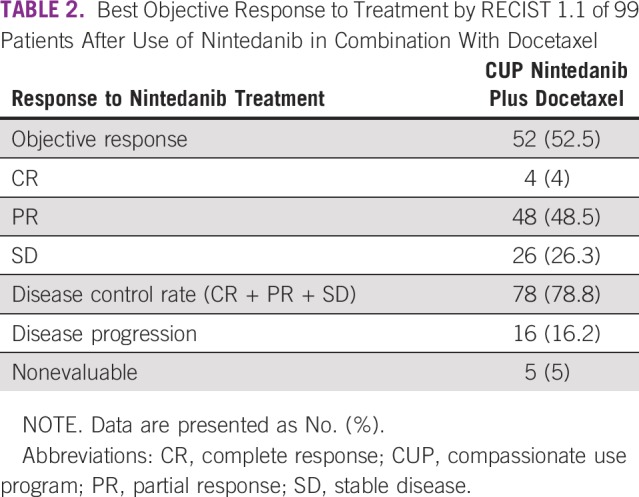
Best Objective Response to Treatment by RECIST 1.1 of 99 Patients After Use of Nintedanib in Combination With Docetaxel

**TABLE 3:**
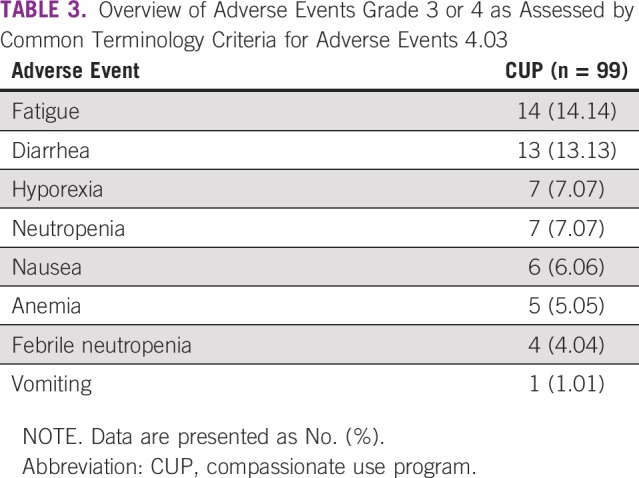
Overview of Adverse Events Grade 3 or 4 as Assessed by Common Terminology Criteria for Adverse Events 4.03

**FIG 3:**
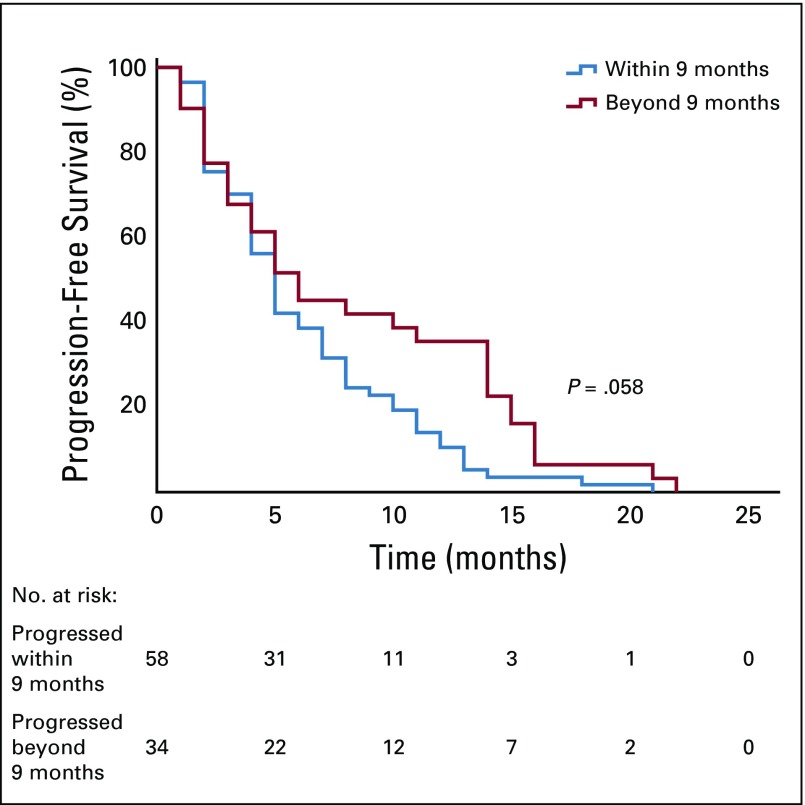
Progression-free survival within 9 months and beyond 9 months.

Exploratory analysis of patients with rapidly progressive disease (defined as a progression within 9 months after first-line treatment)^5^ reported a total of 58 rapid progressors and 34 patients who progressed beyond 9 months after first-line treatment. The median PFS shown in Fig 3 of patients who progressed within 9 months was 5 months (95% CI, 4.2 to 5.8 months) versus a median PFS of 6 months from those who progressed beyond 9 months (95% CI, 1.9 to 10.1 months; *P* = .058). After bivariate analysis between those with rapidly progressive disease and those without, no statistical difference was found in the comparison of median duration of treatment with nintedanib (*P* = .835), the best response to nintedanib (*P* = .100), or disease control rate (complete response + partial response + stable disease; *P* = .12).

## DISCUSSION

The general characteristics of the population are consistent with previous reports described in the literature.^5,9,28,29^ The average age was 60 years (range, 29-84 years), and stage IV was the most prevalent clinical stage at the beginning of the compassionate program. The current study allowed the inclusion of patients who had received antiangiogenic agents, other than nintedanib, as part of their first-line treatments, of whom only 7% had received bevacizumab as a first-line agent and 38% had received maintenance therapy. This proves relevant because both strategies increased both the PFS and the OS of patients. Greater response rates than those obtained in LUME-Lung 1 were obtained in this study,^5^ which could be explained by the high prevalence of patients without previous exposure to antiangiogenic drugs.^35^ Nonetheless, the retrospective nature of this study and the heterogeneous evaluation of each researcher is an important variable to consider. The PFS in the current study was similar to values reported in previous studies,^5,29^ giving greater validity to nintedanib as an effective second-line treatment option in a real-life setting.

Although traditionally, patients with a better PFS and OS after docetaxel with nintedanib were those with rapidly progressive disease,^5^ this study did not find any statistical difference between this group and the group without rapidly progressive disease. This is relevant because immunotherapy as a second-line treatment has shown an attenuated efficacy in patients with a negative PD-L1, in those with rapidly progressive disease, in women, and in nonsmokers.^32,36^

The toxicity profile of this study reported less adverse events and an adequate tolerance to treatment. Diarrhea, neutropenia, and febrile neutropenia were seen less frequently than reported in the literature, which is probably because of the retrospective nature of the study. In this study, oncologists were constantly aware of reported toxicity profiles, which may have helped them prevent and treat these scenarios more efficiently.^5,24,25^

The LUME-Lung 1 trial demonstrated a benefit not only in PFS, but also in OS in patients treated with docetaxel plus nintedanib when compared with nintedanib alone as second-line treatment in advanced lung adenocarcinoma, with a disease progression of less than 9 months. Because of the retrospective nature of our study, limitations in collecting OS rates were found.

Finally, it is important to consider that second-line treatment of wild-type lung adenocarcinoma has changed dramatically since the approval of immunotherapies (anti-PD1, anti–PD-L1) such as nivolumab, pembrolizumab, and atezolizumab, increasing OS when compared with docetaxel alone. However, these studies do not include the use of second-line–approved antiangiogenic agents such as nintedanib or ramucirumab in combination with docetaxel, and although immunotherapies have shown benefit to those with some degree of expression of PD-L1, the same benefit is not so clear for those with a PD-L1–negative tumor; therefore, additional investigation is required.

The previous results were extracted from Mexican patients included in the compassionate use program with nintedanib in a real-life setting. In Mexico, access to oncologic treatment has multiple limitations including economic, sociodemographic, and health system access issues, hindering access to novel treatments. The advantages of participating in a compassionate program are invaluable, not only because patients can receive novel treatments, but also because it increases oncologist expertise in the management of agent toxicity, which generates new information for the management of populations with similar ethnic profiles and provides greater insight for future research.

Nintedanib, as part of a chemotherapy regimen, is an effective option for advanced lung adenocarcinoma after first-line treatment progression; however, additional investigation making direct comparisons with immunotherapy should be performed, taking into account a wider oncologic profile, such as the expression of PD-L1.
